# Cross-Linked Crystals of Dirhodium Tetraacetate/RNase A Adduct Can Be Used as Heterogeneous Catalysts

**DOI:** 10.1021/acs.inorgchem.3c00852

**Published:** 2023-05-05

**Authors:** Domenico Loreto, Basudev Maity, Taiki Morita, Hiroyuki Nakamura, Antonello Merlino, Takafumi Ueno

**Affiliations:** † Department of Chemical Sciences, University of Naples Federico II, Napoli I-80126, Italy; ‡ School of Life Science and Technology, Tokyo Institute of Technology, 4259-B55 Nagatsuta-cho, Midori-ku, Yokohama 226-8501, Japan; § Laboratory for Chemistry and Life Science, Institute of Innovative Research, Tokyo Institute of Technology, 4259 Nagatsuta-cho, Midori-ku, Yokohama 226-8503, Japan; ∥ Living Systems Materialogy Research Group, International Research Frontiers Initiative, Tokyo Institute of Technology, Yokohama 226-8501, Japan

## Abstract

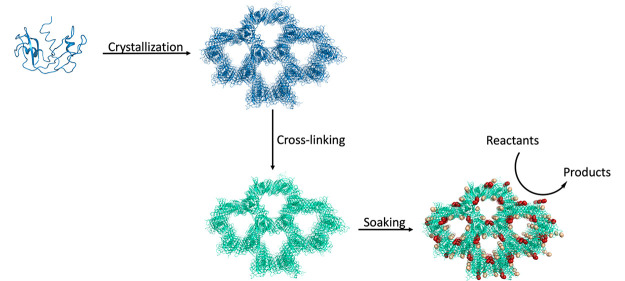

Due to their unique coordination structure, dirhodium paddlewheel complexes are of interest in several research fields, like medicinal chemistry, catalysis, etc. Previously, these complexes were conjugated to proteins and peptides for developing artificial metalloenzymes as homogeneous catalysts. Fixation of dirhodium complexes into protein crystals is interesting to develop heterogeneous catalysts. Porous solvent channels present in protein crystals can benefit the activity by increasing the probability of substrate collisions at the catalytic Rh binding sites. Toward this goal, the present work describes the use of bovine pancreatic ribonuclease (RNase A) crystals with a pore size of 4 nm (*P*3_2_21 space group) for fixing [Rh_2_(OAc)_4_] and developing a heterogeneous catalyst to perform reactions in an aqueous medium. The structure of the [Rh_2_(OAc)_4_]/RNase A adduct was investigated by X-ray crystallography: the metal complex structure remains unperturbed upon protein binding. Using a number of crystal structures, metal complex accumulation over time, within the RNase A crystals, and structures at variable temperatures were evaluated. We also report the large-scale preparation of microcrystals (∼10–20 μm) of the [Rh_2_(OAc)_4_]/RNase A adduct and cross-linking reaction with glutaraldehyde. The catalytic olefin cyclopropanation reaction and self-coupling of diazo compounds by these cross-linked [Rh_2_(OAc)_4_]/RNase A crystals were demonstrated. The results of this work reveal that these systems can be used as heterogeneous catalysts to promote reactions in aqueous solution. Overall, our findings demonstrate that the dirhodium paddlewheel complexes can be fixed in porous biomolecule crystals, like those of RNase A, to prepare biohybrid materials for catalytic applications.

## Abstract 2

Porous RNase A crystals were stabilized by cross-linking and functionalized with dirhodium tetraacetate. The metal/protein adduct was extensively characterized by X-ray crystallography. Cross-linked crystals of the metal/protein adduct were used as heterogeneous catalysts for reactions in aqueous solutions.

## Introduction

Protein crystals are useful materials for structural analysis.^1^ They consist of protein units repeated in a long-range 3D order, which interact with each other by weak interactions.^2^ This ordered architecture is characterized by the presence of large solvent channels, which make the protein crystal lattice porous (solvent channels occupy 30–60% of the whole crystal volume).^3^ Solvent channels are large enough to allow the diffusion of exogeneous molecules within the crystal. The study of the interaction of proteins with organic molecules, metal ions, or complexes has provided insightful information on the binding mechanism of these ligands to proteins.^4−6^

However, the weak interactions between symmetry mates and the large pores that are formed within protein crystals make these systems very fragile and unstable when they are removed from their mother liquor, limiting their applications as biohybrid materials.^7^ This drawback can be overcome using the covalent cross-linking strategy, which allows the formation of cross-linked protein crystals (CLPCs). CLPCs are stable and insoluble in both organic and aqueous solvents and display good mechanical and thermal stability.^8,9^ CLPCs are usually prepared using glutaraldehyde (GA) as the cross-linking agent because both monomeric and oligomeric GA species can interact with proteins,^10−12^ making the cross-linking process nonspecific and thus useful for linking crystals of different proteins, regardless the nature of the protein or crystal packing.^7^ CLPCs have been used for several applications ranging from biosensing to drug delivery.^13−17^ CLPCs are of great interest for the preparation of heterogeneous catalysts based on artificial metalloenzymes.^18^ In fact, when a protein crystal is stabilized by cross-linking, metal complexes can diffuse through solvent channels of the crystal lattice and interact with protein molecules, forming a stable metal/protein adduct crystal.^19,20^ This system can be used as an effective heterogeneous catalyst because it allows one to screen better reaction conditions for catalysis. Indeed, it can tolerate the presence of high concentrations of organic compounds in aqueous solvents and thermal and pH variations.^8,9^ Moreover, CLPCs overcome the limitation of both metal complexes and proteins alone. In fact, in CLPCs, the metal first, second, and third coordination spheres can be modulated by protein engineering, providing a catalyst with unprecedented performances,^18^ while the protein can retain its folding within the crystal lattice, even at extreme pH or in the presence of a high percentage of organic solvents. Hence, these systems can, in principle, improve the performances not only of the metal complexes but also of artificial metalloenzymes. Some of us have functionalized cross-linked hen egg white lysozyme (HEWL) (CL_HEWL) crystals with [Ru(benzene)Cl_2_]_2_ and have tested the reactivity of cross-linked crystals of this metal/protein adduct toward transfer hydrogenation of acetophenone derivatives.^21^ CLPCs catalyzed the transfer hydrogenation reaction better than the metal/protein adduct in solution.^21^ CL_HEWL crystals have also been used for growing platinum nanoparticles (from H_2_PtCl_6_), useful as hydrogen evolution promoters.^22^ CLPC-based catalysts can be recycled several times before inactivation.^22^ Cross-linked NikA (a nickel binding protein) crystals have been functionalized with Fe-EDTA-like compounds (EDTA = ethylenediaminotetraacetate) and employed as oxidation catalysts for C–C double bond, using O_2_ as an oxidant.^23^

Despite the tremendous advantages that arise from the metal functionalization of CLPCs, those mentioned above are the only known examples of the use of these systems as catalysts.

**Figure 1:**
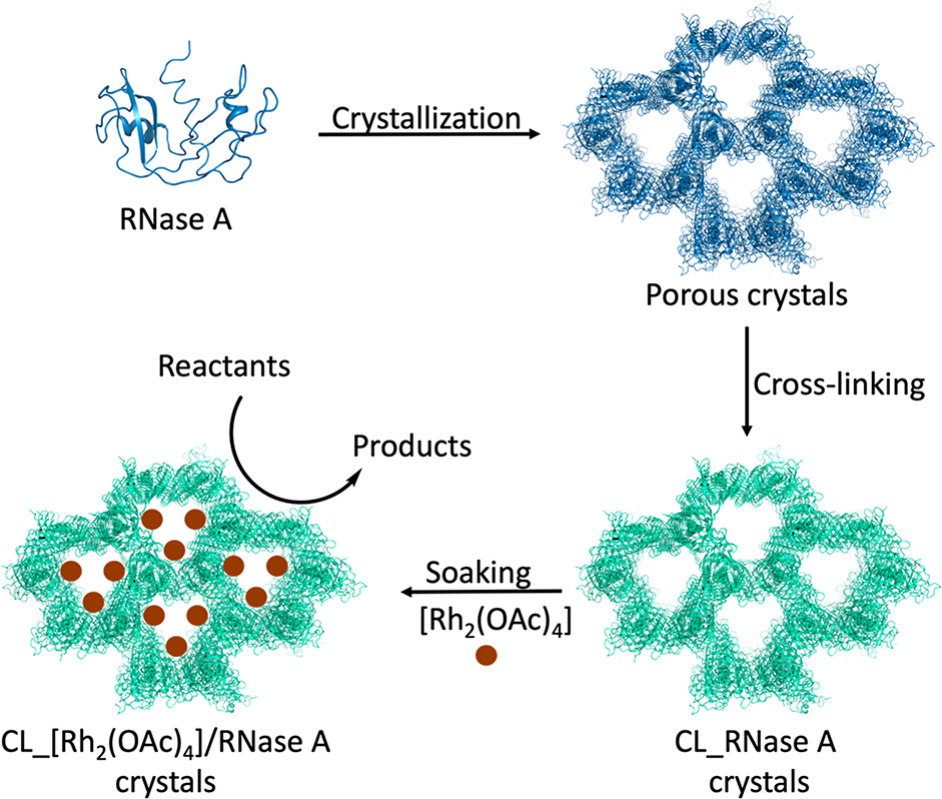
Schematic illustration of the preparation and use of cross-linked crystals of the [Rh_2_(OAc)_4_]/RNase A adduct as catalysts. The protein was crystallized; protein crystals were cross-linked using GA and soaked with dirhodium tetraacetate to prepare biohybrid heterogeneous catalysts.

Dirhodium tetracarboxylates [Rh_2_(μ-O_2_CR)_4_L_2_] (R = generic organic chain; L = electron donor ligand) are versatile catalysts for several reactions,^24,25^ like decomposition of diazo compounds,^26^ carbene insertion into aliphatic and aromatic C–H bonds,^27,28^ nitrene transfer reaction,^29^ olefine cyclopropanation,^30^ aromatic cycloaddition,^31^ hydrosilylation of alkynes,^32,33^ oxidation of alcohols,^34^ and photochemical and thermal hydrogen evolution.^35,36^ Bioconjugation of these complexes with peptides and proteins has provided artificial metalloenzymes useful for stereoselective C–H insertion reaction and hydrosilylation,^37,38^*inter*molecular cyclopropanation,^39−41^ and selective (*E*)-alkene coupling.^42^ Some of us have previously studied the interaction of these complexes with proteins by X-ray crystallography,^43−47^ revealing that they degrade within HEWL crystals,^44,46,47^ while they react with bovine pancreatic ribonuclease (RNase A) in the solid state, forming a metal/protein adduct where the dirhodium core remains intact.^45^ Crystals of the [Rh_2_(OAc)_4_]/RNase A adduct (OAc = acetate) belonged to the *C*2 space group, with two protein molecules in the asymmetric unit. In both protein molecules, two dirhodium binding sites were observed, in the proximity of His105 and His119 side chains. In both cases, the dimetallic paddlewheel scaffold is preserved and Rh atoms are linked to the N atom of the His imidazole rings at the axial position. The interaction of [Rh_2_(OAc)_4_] with RNase A does not suppress the reactivity of the dimetallic compound toward small ligands, like imidazole, in the solid state.^48^ Hence, [Rh_2_(OAc)_4_]/RNase A adduct crystals seem to be ideal candidates for preparing heterogeneous catalysts based on CLPCs. However, crystals of RNase A used for structural determination of the [Rh_2_(OAc)_4_]/RNase A adduct grow in 2 weeks and need one additional week for the adduct formation by soaking.^45^ For this reason, RNase A crystals obtained in other experimental conditions and belonging to another space group (*P*3_2_21) were prepared here. The protocol for preparing good-quality cross-linked crystals of the [Rh_2_(OAc)_4_]/RNase A adduct in this space group was optimized, and cross-linked [Rh_2_(OAc)_4_]/RNase A adduct crystals were prepared, characterized by X-ray crystallography, and tested for the first time as heterogeneous catalysts toward olefin cyclopropanation and self-coupling of the diazo compound reactions (Figure 1). This is the first report of the use of metal/RNase A adduct crystals as functional materials. The results support the idea that the preparation of metal-functionalized CLPCs can be of great interest for developing new biohybrid heterogeneous catalysts.

## Results and Discussion

### Preparation of the [Rh_2_(OAc)_4_]/RNase A Adduct and Investigation of the [Rh_2_(OAc)_4_] Accumulation Process

We chose RNase A crystals from space group *P*3_2_21 to prepare the [Rh_2_(OAc)_4_]/RNase A adduct because they have pores of 40.2 Å (Figure S1A). This pore size is advantageous for the diffusion of small molecules into the crystals. Hence, to prepare more effective systems to be used as heterogeneous catalysts, RNase A crystals were grown under different experimental conditions. An efficient system was obtained by growing RNase A crystals in 2.5 M NaCl, 3.3 M sodium formate, and 0.1 M sodium acetate buffer at pH 4.7–5.5,^49^ using the hanging-drop vapor diffusion technique. Under these conditions, RNase A crystallizes with one protein molecule in the asymmetric unit in 1 day. This crystal form shows wider pores than those found in crystals obtained in the *C*2 space group (Figure S1B).

**Figure 2:**
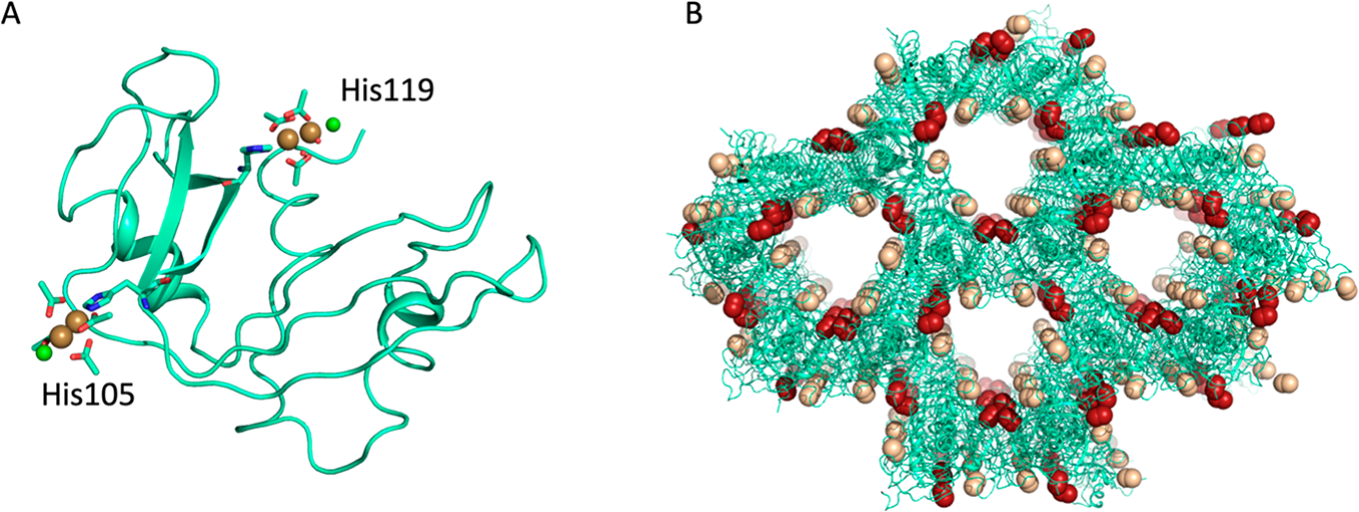
(A) Overall structure of the [Rh_2_(OAc)_4_]/RNase A adduct in *P*3_2_21 crystals (2 h soaking). [Rh_2_(OAc)_4_] binding sites close to the side chains of His105 and His119 are evidenced. (B) Location of Rh atoms in the crystal. Rh atoms close to His105 and His119 are colored in yellow and red, respectively.

It was verified that these crystals can be used to form metal/protein adducts upon reaction with metal compounds in the solid state. Crystals of the adduct with [Rh_2_(OAc)_4_] were formed via soaking^1^ using RNase A crystals grown at pH 5.2. Soaking was performed using a saturated solution of [Rh_2_(OAc)_4_] dissolved in the reservoir. Crystals turned into light pink after 1 h of soaking with the metal compound. To characterize the metal binding process within the protein crystals, X-ray diffraction data were collected on RNase A crystals exposed to [Rh_2_(OAc)_4_] for different soaking times (1, 2, and 6 h). Hence, three different structures of the [Rh_2_(OAc)_4_]/RNase A adduct in the *P*3_2_21 space group were solved (Figure 2A,B). Data collection and refinement statistics are reported in Table 1. Cα root-mean-square deviation (rmsd) resulting from the superimposition of the three structures and that of the metal-free RNase A revealed that they are quite similar to each other and that the metal binding does not alter the overall protein conformation (Table S1), as observed for the structure of the metal/protein adduct derived from crystals in the *C*2 space group.^45^

**Table 1:** Data Collection and Refinement Statistics of the [Rh_2_(OAc)_4_]/RNase A Adduct from Crystals in the *P*3_2_21 Space Group Collected upon Different Soaking Times in the Absence of GA and at Two Different Temperatures in the Presence of GA

	[Rh_2_(OAc)_4_]/RNase A	[Rh_2_(OAc)_4_]/RNase A	[Rh_2_(OAc)_4_]/RNase A	CL_[Rh_2_(OAc)_4_]/RNase A	CL_[Rh_2_(OAc)_4_]/RNase A
soaking time of [Rh_2_(OAc)_4_] (h)	1	2	6	overnight	overnight
soaking time of GA				2	2
temperature (°C)	–180	–180	–180	–180	0
PDB code	8OQC	8OQD	8OQE	8OQF	8OQG
Data Collection					
space group	*P*3_2_21	*P*3_2_21	*P*3_2_21	*P*3_2_21	*P*3_2_21
*a* = *b* (Å)	67.13	67.14	66.79	67.39	67.28
*c* (Å)	64.98	64.79	64.90	64.42	65.90
resolution range (Å)	21.97–1.50	21.98–1.54	21.86–1.50	22.06–1.50	22.02–1.60
	(1.53–1.50)	(1.56–1.54)	(1.53–1.50)	(1.53–1.50)	(1.63–1.60)
unique reflns	27554 (1345)	25583 (1242)	27269 (1349)	27241 (1303)	23142 (1111)
completeness (%)	100.0 (100.0)	100.0 (99.8)	100.0 (99.8)	99.3 (97.6)	99.9 (99.2)
redundancy	11.1 (6.8)	4.1 (2.6)	8.5 (5.1)	1.9 (1.8)	1.8 (1.8)
Rmerge (%)	0.074 (2.144)	0.077 (2.388)	0.079 (2.022)	0.056 (1.296)	0.079 (1.062)
Rpim	0.022 (0.875)	0.041 (1.763)	0.028 (0.975)	0.056 (1.296)	0.079 (1.062)
average *I*/σ(*I*)	22.6 (0.9)	12.3 (0.4)	20.0 (0.8)	17.4 (0.6)	6.9 (0.6)
CC_1/2_	0.999 (0.325)	0.998 (0.087)	0.999 (0.223)	0.998 (0.199)	0.994 (0.339)
anom. completeness (%)	99.8 (98.7)	89.3 (81.6)	99.8 (97.3)	90.5 (86.3)	89.3 (81.6)
anom. redundancy	5.6 (3.4)	1.9 (1.4)	4.2 (2.6)	1.0 (1.0)	0.9 (1.0)
Refinement					
resolution range (Å)	21.98–1.50	21.65–1.54	21.60–1.50	22.06–1.50	22.02–1.60
no. of reflns	26141	24258	25873	25847	21978
*R* factor/*R* free (%)	18.0/21.8	19.6/22.2	17.8/21.5	18.3/22.3	18.1/19.8
no. of atoms	1290	1271	1253	1331	1207
average *B* factors (Å^2^)	20.1	22.0	18.8	19.5	21.2
Rh atoms occupancy	0.75/0.75	0.70/0.70	0.78/0.78	0.75/0.75	0.80/0.80
	0.50/0.50	0.50/0.50	0.55/0.55	0.35/0.35	0.35/0.35
*B* factors of Rh atoms (Å^2^)	14.2/15.5	16.4/17.8	12.6/13.9	15.4/17.0	13.5/15.7
	23.9/28.3	21.1/37.3	24.1/29.5	51.6/59.0	46.3/50.6
Root-Mean-Square Deviations					
bond lengths (Å)	0.010	0.008	0.010	0.009	0.009
bond angles (deg)	1.71	1.48	1.70	1.55	1.55
Ramachandran Statistics (Coot Analysis)					
no. of residues in allowed/disallowed regions	0/0	2/0	1/2	1/0	1/2

In all of the structures, two binding sites for the metal compound were observed, close to His105 and His119 side chains, as observed in previous reports.^45^ These findings indicate that the crystal packing does not influence the binding of the metal compound to the protein. Rh atoms are located on the pore surface and thus in positions that are potentially useful for catalysis. Details of the dirhodium moiety geometry are reported in Table S2 and compared with the values obtained for the [Rh_2_(OAc)_4_]/RNase A adduct in the *C*2 space group .

**Figure 3:**
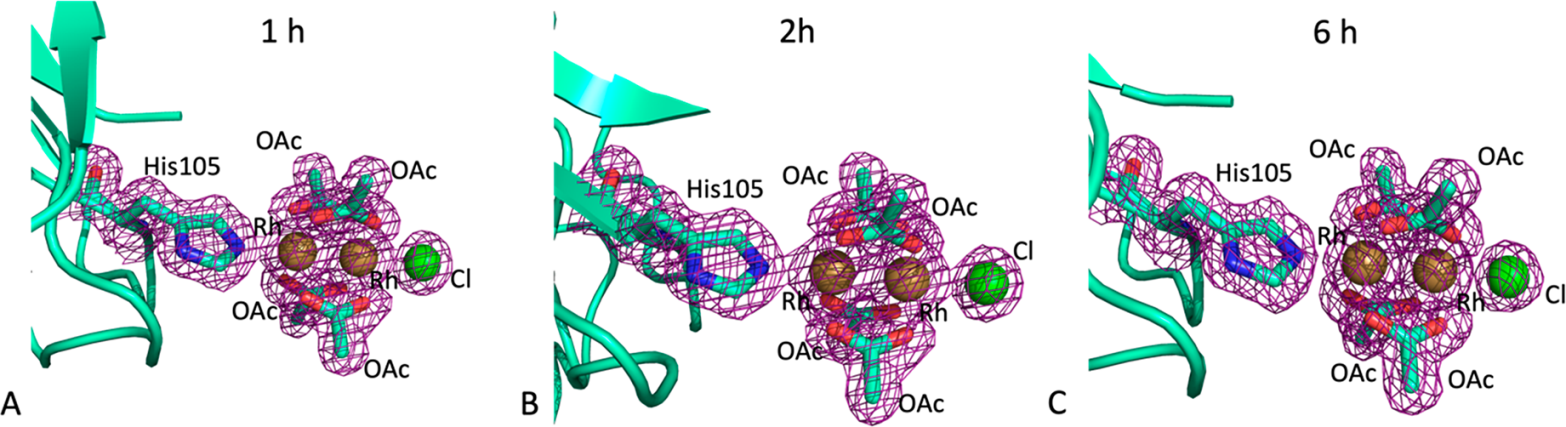
Time-dependent formation of the [Rh_2_(OAc)_4_]/RNase A adduct. Dirhodium binding site is close to the His105 side chain after 1 h (panel A), 2 h (panel B), and 6 h (panel C) of soaking of the RNase A crystals in the reservoir solution containing [Rh_2_(OAc)_4_]. 2*F*_o_ – *F*_c_ electron density maps are contoured at 1.0σ and reported in violet. Cl atoms are in green, and Rh atoms are in yellow.

Close to the His105 side chain, [Rh_2_(OAc)_4_] (occupancy = 0.70–0.78) is linked to the N_ε_ atom of the imidazole ring and to a Cl^–^ ion (Figure 3A–C) through its axial positions. The axial binding of the Cl^–^ ion to the dirhodium core is not surprising considering the high concentration of NaCl in the crystallization conditions.

**Figure 4:**
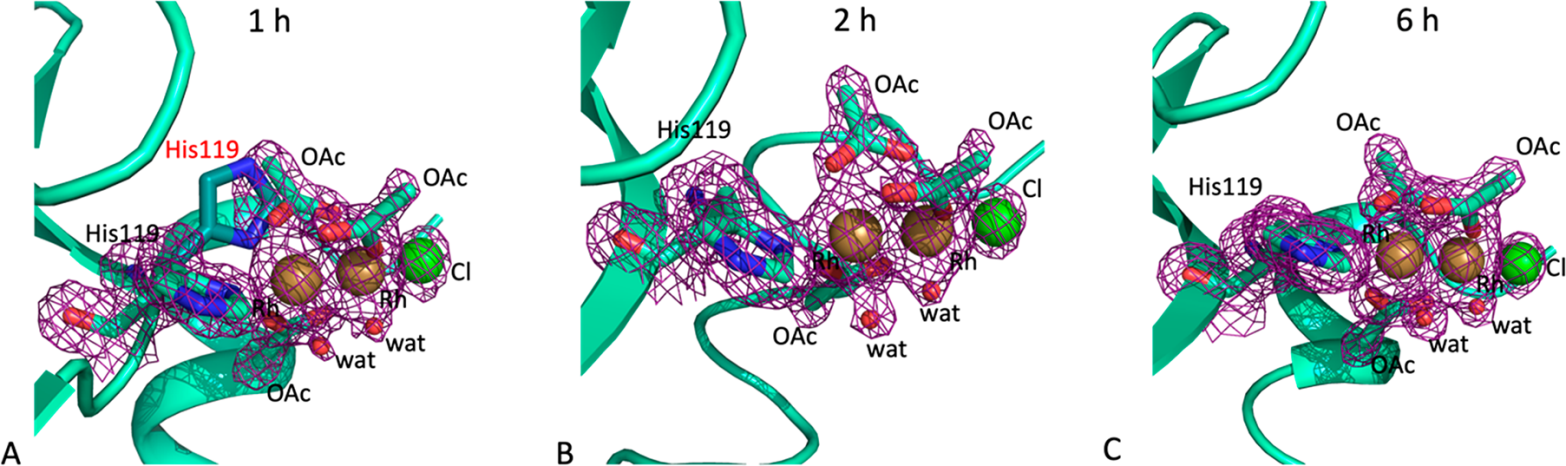
Time-dependent formation of the [Rh_2_(OAc)_4_]/RNase A adduct. Dirhodium binding site close to the His119 side chain after 1 h (panel A), 2 h (panel B), and 6 h (panel C) of soaking of the RNase A crystals in the reservoir solution containing [Rh_2_(OAc)_4_]. 2*F*_o_ – *F*_c_ electron density maps are contoured at 1.0σ and reported in violet. Cl atoms are in green, and Rh atoms are in yellow. The label in red refers to the alternate conformation of His119.

A Cl^–^ ion is found at the axial position of the dirhodium center also in the case of the metal-containing fragment bound to the His119 side chain (Figure 4A–C). However, at this site, the remaining axial position of the dirhodium core (occupancy = 0.50–0.55) is coordinated to the N_δ_ atom of the His imidazole ring, and the dimetallic compound is equatorially coordinated by three acetate ligands and two water molecules. In the structure collected after 1 h of soaking, a double conformation of the His119 side chain is observed (Figure 4A). In conclusion, considering that the occupancy of dirhodium does not significantly change between 1 and 6 h of soaking, it can be assumed that 1 h of soaking is sufficient to metalate the protein in the solid state.

### Preparation of Cross-Linked Crystals of the [Rh_2_(OAc)_4_]/RNase A Adduct

To use crystals of the [Rh_2_(OAc)_4_]/RNase A adduct as catalysts, they should be stabilized by cross-linking. To prepare cross-linked crystals of the [Rh_2_(OAc)_4_]/RNase A adduct (CL_[Rh_2_(OAc)_4_]/RNase A crystals), cross-linked RNase A (CL_RNase A) crystals were prepared and then treated with [Rh_2_(OAc)_4_]. Using this procedure, RNase A crystals resist both soaking and cross-linking treatment. A screening of the GA percentage and cross-linking time was carried out to obtain CL_RNase A crystals with enhanced mechanical properties. The gentle cross-linking technique^50^ was used: GA was added to the reservoir solution, so that it can reach the crystals by vapor diffusion.^50^ CL_RNase A crystals were obtained by exposing protein crystals for 2 h to the reservoir enriched with 0.5% GA. After the cross-linking treatment, few cracks were observed on the lattice surface, but crystals still diffract X-rays and were insoluble in aqueous solutions different from their mother liquor (Figure S2). It is worth noting that when RNase A crystals are exposed to GA vapors for more than 2 h, they lose their diffraction power.

CL_[Rh_2_(OAc)_4_]/RNase A crystals were then prepared. They were obtained by soaking CL_RNase A crystals in a saturated solution of [Rh_2_(OAc)_4_] dissolved in the reservoir overnight. A soaking time higher than 1 h was used to prepare CL_[Rh_2_(OAc)_4_]/RNase A crystals because it is expected that cross-linking could, at least in part, slow down the diffusion of ligands within crystal channels.

The X-ray diffraction data on CL_[Rh_2_(OAc)_4_]/RNase A crystals were collected at two different temperatures, −180 and 0 °C, to evaluate if the temperature could have an effect on the structure of the adduct, when crystals were treated with GA. The two structures solved using data collected at −180 and 0 °C refine at 1.5 and 1.6 Å resolution, respectively. Data collection and refinement statistics are reported in Table 1. The two structures are very similar to each other and to the metal-free structure. C_α_ rmsd analysis revealed that cross-linking does not alter the overall protein structure (Table S1).

**Figure 5:**
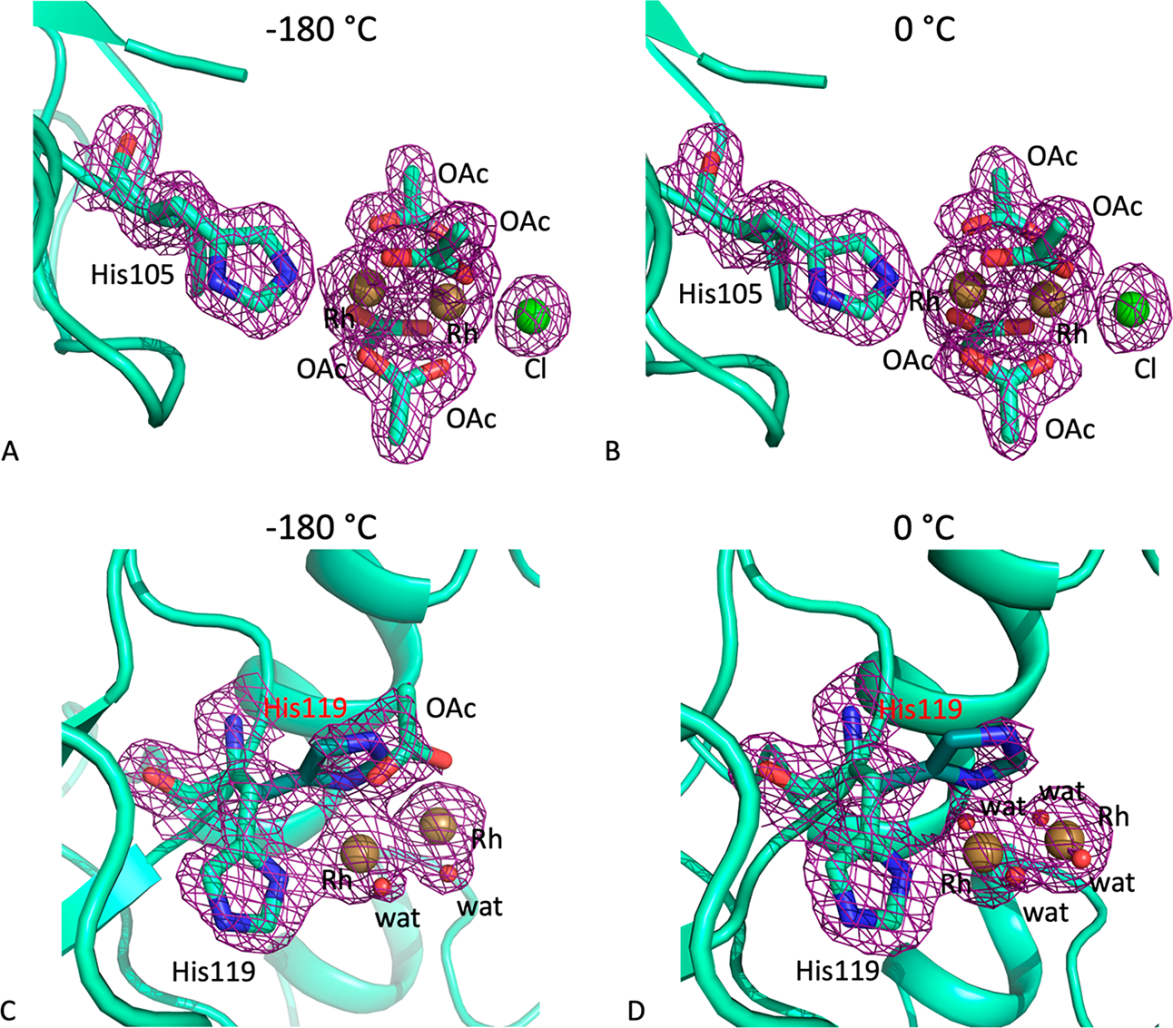
Dirhodium binding sites close to His105 and His119 in the CL_[Rh_2_(OAc)_4_]/RNase A adduct crystals at −180 °C (panels A and C) and 0 °C (panels B and D). 2*F*_o_ – *F*_c_ electron density maps at 1.0σ level are reported in violet. Labels in red indicate alternate conformations of His119 residues. Cl atoms are in green, and Rh atoms are in yellow.

Also in these crystals, two dirhodium binding sites were found, close to the His105 (Figure 5A,B) and His119 (Figure 5C,D) side chains. In both structures, [Rh_2_(OAc)_4_] is linked to the protein via axial binding to the His105 side chain, with high occupancy (0.75–0.80). The remaining axial positions are occupied by a Cl^–^ ion (Figure 5A,B). On the contrary, slightly different results were found close to the His119 side chain. In both structures, the His119 side chain adopts two different conformations, one involved in an axial binding with the dirhodium core and the other perpendicular to the Rh–Rh axis (Figure 5C,D). The low occupancy of the metal complex at this binding site (0.35 in both structures) hampers a clear interpretation of the electron density maps. Nevertheless, in the structure obtained from data collection at −180 °C, one acetate ligand and two water molecules were modeled in the electron density; the other ligands were missing. In the structure derived from the data recorded at 0 °C, only four water molecules could be modeled around the Rh–Rh core at the equatorial position; the other ligands were missing. This result could suggest a more extensive hydrolysis of the dimetallic complex when the metal protein adduct is exposed to X-ray at higher temperature.

A comparison of the results obtained from crystals of the [Rh_2_(OAc)_4_]/RNase A adduct formed in the absence and in the presence of GA indicates that the binding of [Rh_2_(OAc)_4_] to the protein close to the His105 side chain is not affected by the cross-linking, while a decrease of the occupancy is observed for the dirhodium center bound to the His119 side chain, upon treatment of the crystals with GA.

Overall, these results reveal that CL_RNase A crystals can be metalated by [Rh_2_(OAc)_4_] using a soaking technique and that the structure of the adduct is unaffected by the data collection temperature.

### Use of CL_[Rh_2_(OAc)_4_]/RNase A Crystals as Catalysts

Catalysis requires CLPCs in bulk scale that cannot be obtained by a hanging-drop vapor diffusion technique. Hence, RNase A crystals were grown via the batch technique in 2.5 M NaCl, 3.3 M sodium formate, and 0.1 M sodium acetate buffer at pH 5.2. The smaller size of these crystals compared to those obtained using the hanging-drop vapor diffusion method is useful for catalysis because small crystals allow better substrate diffusion toward the catalytic center. These crystals were treated with GA and functionalized with [Rh_2_(OAc)_4_] (see Methods for details and Figure S3).

**Scheme 1:**
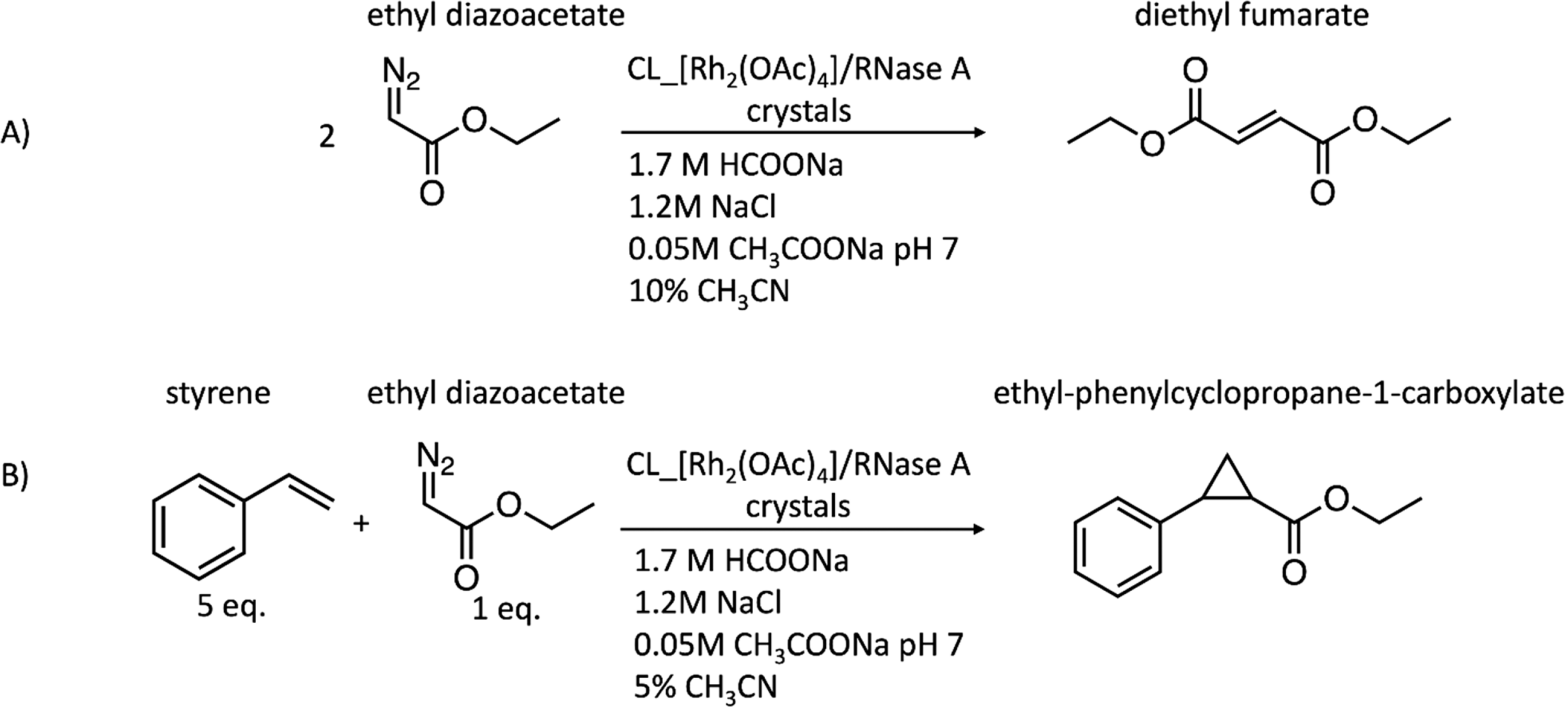
Scheme Showing Self-Coupling of the Diazo Compound (Panel A) and Olefin Cyclopropanation (Panel B) Reactions Used for the Catalysis by CL_[Rh_2_(OAc)_4_]/RNase A Crystals

The ability of CL_[Rh_2_(OAc)_4_]/RNase A crystals as heterogeneous catalysts toward self-coupling of diazo compounds and olefin cyclopropanation reactions was assayed (Scheme 1). The reactions were performed in an aqueous solvent at 4 °C, a temperature close to that used for structural analysis of the CL_[Rh_2_(OAc)_4_]/RNase A crystals.

Analysis of the reaction products by gas chromatography (GC)–mass spectrometry (MS) indicated that both reactions were promoted by CL_[Rh_2_(OAc)_4_]/RNase A crystals (Figures S4 and 6). MS analysis of the chromatogram derived from the reaction products of the self-coupling reaction revealed the presence of the coupling product, diethyl fumarate at *t*_R_ = 9.63 min (conversion = 2.6%). Also, its hydrolysis derivative was found at *t*_R_ = 9.34 min (Table 2). Although the GC–MS elution profile revealed other side reactions, the presence of diethyl fumarate at 9.63 min for diazo coupling suggests that the CL_[Rh_2_(OAc)_4_]/RNase A crystals can be used as heterogeneous catalysts (Figure S4).

**Table 2:** Retention Time (*t*_R_), Theoretical Molecular Weight, and Experimental Mass of the Molecular Radical Cation of the Reactants and Products Obtained upon Styrene Cyclopropanation and Ethyl Diazoacetate Self-Coupling Reactions Catalyzed by CL_[Rh_2_(OAc)_4_]/RNase A Crystals

chemical	*t*_R_ (min)	molecular weight (Da)	mass of the molecular radical cation (*m*/*z*)
styrene	4.88	104.15	104
ethyl diazoacetate	4.33	114.10	114
(*E*)-4-ethoxy-4-oxobut-2-enoic acid (hydrolysis product of diethyl fumarate)	9.34	144.04	n.o. (bp 99)^a^
diethyl fumarate	9.63	172.18	n.o. (bp 127)^a^
*cis*-ethyl phenylcyclopropane-1-carboxylate	13.38	190.24	190
*trans*-ethyl phenylcyclopropane-1-carboxylate	14.08	190.24	190

a n.o. = not observed. bp = base peak.

**Figure 6:**
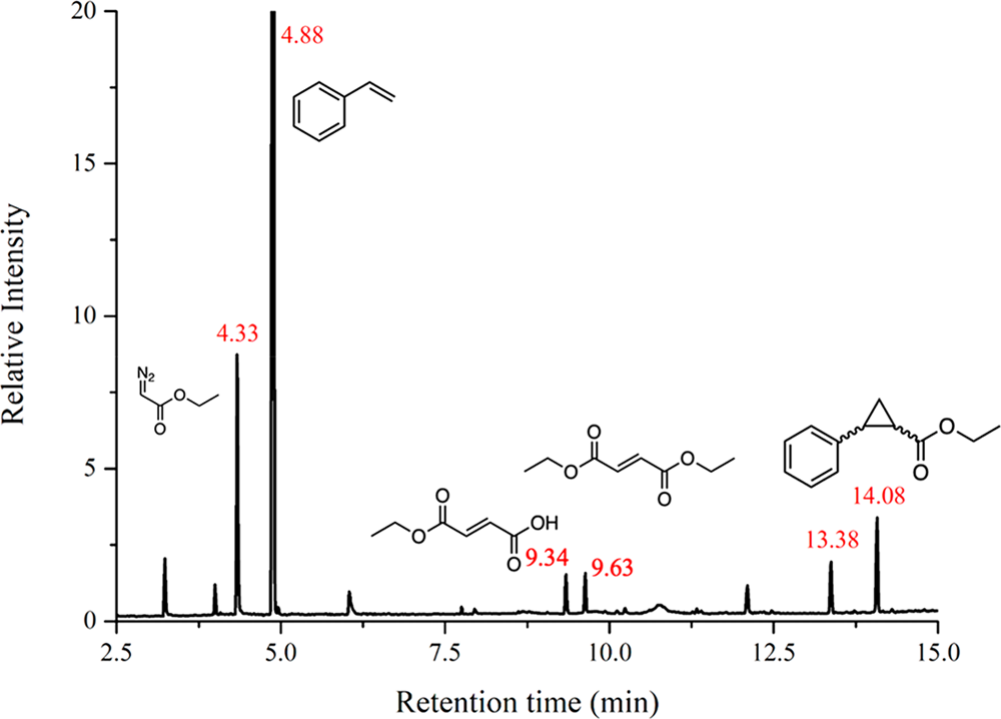
GC chromatogram of the reaction products obtained upon the olefin cyclopropanation reaction catalyzed by CL_[Rh_2_(OAc)_4_]/RNase A crystals. Labels in red indicate retention times of identified reaction products. The unlabeled peaks were not assigned. The peaks at *t*_R_ = 13.38 and 14.08 min were attributed to the cis and trans isomers of ethyl phenylcyclopropane-1-carboxylate, respectively.

Then, a catalytic styrene cyclopropanation reaction was promoted using CL_[Rh_2_(OAc)_4_]/RNase A crystals. The GC–MS elution profile reported in Figure 6 of the reaction products (*t*_R_ = 13.38 and 14.08 min; Table 2) revealed that styrene is selectively converted into the two cis and trans isomers of ethyl phenylcyclopropane-1-carboxylate (conversion = 55.1%). To confirm the product assignment of the cis and trans isomers of the cyclopropane derivative formed upon styrene cyclopropanation, we performed a control GC–MS run using a synthetically prepared cis isomer (Figure S5). The chromatogram of *cis*-ethyl phenylcyclopropane-1-carboxylate showed a peak at *t*_R_ = 13.38 min, perfectly matching the results derived from the reaction catalyzed by CLCPs. Hence, the peak at *t*_R_ = 14.08 min (Figure 6) can be assigned to the *trans*-ethyl phenylcyclopropane-1-carboxylate. The ratio between these two peaks (cis/trans = 1:2.4) indicates a slight preference of the CL_[Rh_2_(OAc)_4_]/RNase A crystals in promoting formation of the trans isomer rather than the cis isomer.

Control experiments using CL_RNase A crystals as catalysts of the above-mentioned reactions were performed under the same experimental conditions (Figures S6–S8). The results revealed that CLPCs are not efficient to promote these reactions when they have not been treated with the metal catalyst because the self-coupling reaction occurs with a very low conversion (less than 0.5%), while olefin cyclopropanation is not catalyzed at all. Therefore, we can conclude that the porous RNase A crystals functionalized with the metal compound can be used as a heterogeneous catalyst to promote reactions in an aqueous medium.

## Conclusions

In summary, we have prepared [Rh_2_(OAc)_4_]-functionalized cross-linked RNase A crystals considering the 4 nm porous channels present in the *P*3_2_21 space group. Cross-linking treatment was done for stabilization of the crystals and for potential application as heterogeneous catalysts. The structure of the adduct and the dirhodium accumulation mechanism in these crystals over time were investigated via X-ray crystallography. Structural analysis demonstrates that 1 h of soaking is sufficient to metalate the protein and that the crystal packing does not influence the binding of the metal compound to RNase A: two binding sites were identified for the metal compound, close to the His105 and His119 side chains, in agreement with that observed using RNase A crystals in a different crystal form.^45^ These sites are on the pore surface, thus in a position that is potentially useful for catalysis. Cross-linking treatment and structure determination at −180 and 0 °C revealed no significant change, thus suggesting perfect stabilization of Rh in the RNase A scaffold. Thus, CL_[Rh_2_(OAc)_4_]/RNase A crystals were used as heterogeneous catalysts toward self-coupling of the diazo compound and olefin cyclopropanation reactions. Although side reactions were observed, GC–MS analysis of the reaction products revealed that both reactions are promoted by CLPCs functionalized with Rh and that CL_[Rh_2_(OAc)_4_]/RNase A crystals can be used as heterogeneous catalysts in an aqueous medium. To the best of our knowledge, metalated RNase A crystals were used for the first time as heterogeneous catalysts. Due to the presence of a 4 nm porous channel, the current results reveal that the metalation of CLPCs can expand the functionality of a protein beyond its natural one. This could give rise to a new generation of heterogeneous catalysts that take advantage of the synergistic action of the metal complexes, protein scaffold, and crystal lattice.

## Methods

### Materials

All of the chemicals used in this work, including RNase A, were purchased from TCI (Wako, Nacalai Tesque) at the highest degree of purity available and used without further purification. Organic solvents were purchased from Merck Life Science.

### Synthesis of Ethyl Phenylcyclopropane-1-carboxylate

**Scheme 2:**
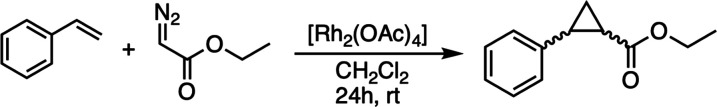
Synthesis of Ethyl Phenylcyclopropane-1-carboxylate

The two isomers of ethyl phenylcyclopropane-1-carboxylate were prepared according to Scheme 2.^51^

A total of 43 mmol (4.5 g) of styrene was dissolved in 5.0 mL of CH_2_Cl_2_, and 6.8 mg of [Rh_2_(OAc)_4_] was added to the solution. A solution of ethyl diazoacetate (68 mmol, 7.8 mg) was added dropwise to the reaction blend at room temperature over 5 h. After 4 h, 1 mg of [Rh_2_(OAc)_4_] was added to the solution, and the reaction mixture was left at room temperature over magnetic stirring for 24 h. Then, the mixture was concentrated under reduced pressure and purified by column chromatography (Merck Kiesgel 60) of the crude residue over silica gel (ethyl acetate/hexane 9:1). *cis*-Ethyl phenylcyclopropane-1-carboxylate was obtained (39.4 g, 48,6% yield) as a pale-yellow oil. ^1^H NMR spectra were coherent with those reported elsewhere (Figure S5).^51^

### Preparation of Crystals of the [Rh_2_(OAc)_4_]/RNase A Adduct

RNase A crystals in the *P*3_2_21 space group were prepared at 20 °C using the hanging-drop vapor diffusion technique. A RNase A solution (50 mg/mL) was equilibrated with a reservoir consisting of 2.5 M NaCl, 3.3 M sodium formate, and 0.1 M sodium acetate buffer at pH 5.2. Crystals were obtained in 1 day. Soaking was performed by transferring protein crystals into a solution consisting of the reservoir saturated with [Rh_2_(OAc)_4_] for 1, 2, or 6 h. Crystals turned into light pink upon exposure to the metal complex.

### Preparation of CL_[Rh_2_(OAc)_4_]/RNase A Adduct Crystals

RNase A crystals (prepared as described above) were washed and equilibrated versus a reservoir consisting of 2.5 M NaCl, 3.3 M sodium formate, and 0.1 M sodium acetate buffer at pH 5.2 enriched with 0.5% GA. After 2 h, crystals turned into pale yellow and CL_RNase A crystals were formed (Figure S2). Crystals were transferred to a solution containing the original reservoir to remove the excess of GA and then soaked with [Rh_2_(OAc)_4_] overnight.

### Preparation of CL_[Rh_2_(OAc)_4_]/RNase A Adduct Crystals with the Batch Technique

A total of 100 μL of RNase A (300 mg/mL) was mixed with 400 μL of a solution consisting of 2.5 M NaCl, 3.3 M sodium formate, and 0.1 M sodium acetate buffer at pH 5.2 (final protein concentration = 60 mg/mL). Upon 24 h, many crystals were obtained. Cross-linking of these crystals was performed by adding 0.1% GA to the solution containing the crystals. After 90 min, yellow CL_RNase A crystals obtained upon this treatment were washed with the reservoir solution and soaked with the same solution saturated with [Rh_2_(OAc)_4_] for 6 h. Upon metalation, crystals turned pink (Figure S3). The excess of [Rh_2_(OAc)_4_] on the surface of the crystals was washed out using the reservoir solution.

### Data Collections, Structure Resolutions, and Refinements

X-ray diffraction data were collected using a Rigaku XtaLAB Synergy-DW diffractometer equipped with a HyPix-6000HE detector at Tokyo Institute of Technology, Yokohama, Japan. Before data collection, crystals of the [Rh_2_(OAc)_4_]/RNase A adduct were flash-frozen using the crystallization buffer containing 30% (v/v) glycerol as a cryoprotectant. No cryoprotectant was used for CLPCs. Diffraction data on crystals of the adducts were collected at −180 °C. Data collection for CPLCs was performed at both −180 and 0 °C. Data were processed and scaled using *Aimless*.^52^ The structures were solved in the *P*3_2_21 space group with a single molecule in the asymmetric unit by a molecular replacement method using *Phaser*.^53^ Metal-free RNase A under the accession code 5OGH was used as starting model.^54^ Restrained refinement and model building were carried out using Refmac5 and Coot, respectively.^55,56^ Anomalous difference electron density maps were used to identify the Rh positions in the electron density maps. The PDB validation server (https://validate-rcsb-2.wwpdb.org) was used for structure validation. The structures were deposited at PDB with accession codes 8OQC, 8OQD, 8OQE, 8OQF, and 8OQG.

### Catalysis of the Olefin Cyclopropanation Reaction

CL_[Rh_2_(OAc)_4_]/RNase A crystals prepared through the batch technique were suspended in 500 μL of a solution consisting of 1.25 M NaCl, 1.75 M sodium formate, and 0.05 M sodium acetate at pH 7.0.

A total of 25 μL of styrene (0.97 M stock solution in acetonitrile) was dissolved in the solution, and then 25 μL of ethyl diazoacetate dissolved in acetonitrile (0.50 M stock solution) was added to the reaction mixture (final concentrations: styrene = 4.4 mM, ethyl diazoacetate = 8.7 mM, and acetonitrile = 10%). The reaction was stirred at 4 °C for 17 h, then quenched with CHCl_3_, and extracted three times with 600 μL of CHCl_3_. The resulting mixture was analyzed by GC–MS. The Rh concentration (0.9 mM) in the reaction mixture was determined by inductively coupled plasma mass spectroscopy (ICP-MS).

### Catalysis of Olefin Self-Coupling of the Diazo Compound Reaction

A total of 15 μL of ethyl diazoacetate (0.50 M stock solution in acetonitrile) was dissolved into a solution consisting of CL_[Rh_2_(OAc)_4_]/RNase A crystals, 1.25 M NaCl, 1.75 M sodium formate, and 0.50 M sodium acetate at pH 7.0 (final concentrations: ethyl diazoacetate = 2.5 mM and acetonitrile = 5%). The reaction was stirred at 4 °C for 17 h. The reaction was quenched using CHCl_3_ and extracted three times with the same solvent. The resulting mixture was analyzed by GC–MS. The Rh concentration (0.9 mM) in the reaction mixture was determined by ICP-MS.

### GC–MS Analysis

The GC–MS data were acquired with a Shimadzu GC-MS QP2010 spectrometer equipped with a Shimadzu SH-Rxi-5Sil column (inner diameter = 0.25 nm; film thickness = 0.25 μm). The temperature was increased from 35 °C up to 320 °C using a gradient of 10 °C/min. The final temperature was maintained for 2 min before cooling. The substrate conversion of the two reactions was calculated as the ratio between the peak areas of the reaction products and reactants. The ratio between the cis and trans isomers of ethyl phenylcyclopropane-1-carboxylate was evaluated by calculating the ratio between their peak areas.

### ICP-MS Measurements

CL_[Rh_2_(OAc)_4_]/RNase A crystals were dissolved in 1 mL of HNO_3_ (70% v/v). The solution was diluted up to 5 mL. A total of 77 μL of this solution was diluted to a final volume of 10 mL. The metal concentration in the solution was determined by ICP-MS (PerkinElmer Japan, ELAN DRC-es). Standard solutions were prepared using Standard Solution *G* purchased from Kanto Chemical Co. (Rh concentration = 10 mg/L).

## Supporting Information Available

Crystal packing in the *P*3_2_21 and *C*2 space groups (Figure S1), images of RNase A crystals grown in 2.5 M NaCl, 3.3 M sodium formate, and 0.1 M sodium acetate at pH 5.2 before and after GA treatment (Figure S2), pictures of bulk RNase A crystals obtained using the batch technique and after soaking with dirhodium tetraacetate (Figure S3), GC chromatogram of the reaction products obtained upon self-coupling of the diazo compound reaction catalyzed by cross-linked crystals of the adduct (Figure S4), ^1^H NMR spectrum of the *cis*-ethyl phenylcyclopropane-1-carboxylate (Figure S5), GC chromatogram used as the control for the olefin cyclopropanation reaction (Figure S6), GC chromatogram used as the control for self-coupling of the diazo compound reaction (Figure S7), GC chromatogram of a styrene and ethyl diazoacetate mixture (Figure S8), Rmsd obtained by the superimposition of C_α_ atoms of the various structures discussed in the paper (Table S1), selected bond lengths and angles for the dirhodium center in the structures reported in this paper (Table S2) (PDF)
