# Supplementary material for: Cross-Linked Crystals of Dirhodium Tetraacetate/RNase A Adduct Can Be Used as Heterogeneous Catalysts
Source: Inorg Chem. 2023 May 5;62(19):7515–24. doi: 10.1021/acs.inorgchem.3c00852 (PMC10189731; doi:10.1021/acs.inorgchem.3c00852)
Supplement: Supplementary file 1 — ic3c00852_si_001.pdf [file ic3c00852_si_001.pdf]

Supporting information

Cross-linked crystals of dirhodium tetraacetate/RNase A adduct can be used as heterogeneous catalysts

Domenico Loreto,[1] Basudev Maity,[2] Taiki Morita,[2,3] Hiroyuki Nakamura,[2,3] Antonello Merlino,[1*] Takafumi Ueno[2,4*]

[1]Department of Chemical Sciences, University of Naples Federico II, I-80126, Napoli, Italy

[2]School of Life Science and Technology, Tokyo Institute of Technology, Nagatsuta-cho 4259-B55, Midori-ku, Yokohama 226-8501, Japan

[3]Laboratory for Chemistry and Life Science, Institute of Innovative Research, Tokyo Institute of Technology, 4259, Nagatsuta-Cho, Midori-Ku, Yokohama, 226-8503, Japan

[4]Living Systems Materialogy (LiSM) Research Group, International Research Frontiers Initiative (IRFI), Tokyo Institute of Technology, Yokohama 226-8501, Japan

Correspondence to Antonello Merlino (antonello.merlino@unina.it) or to Takafumi Ueno (tueno@bio.titech.ac.jp)

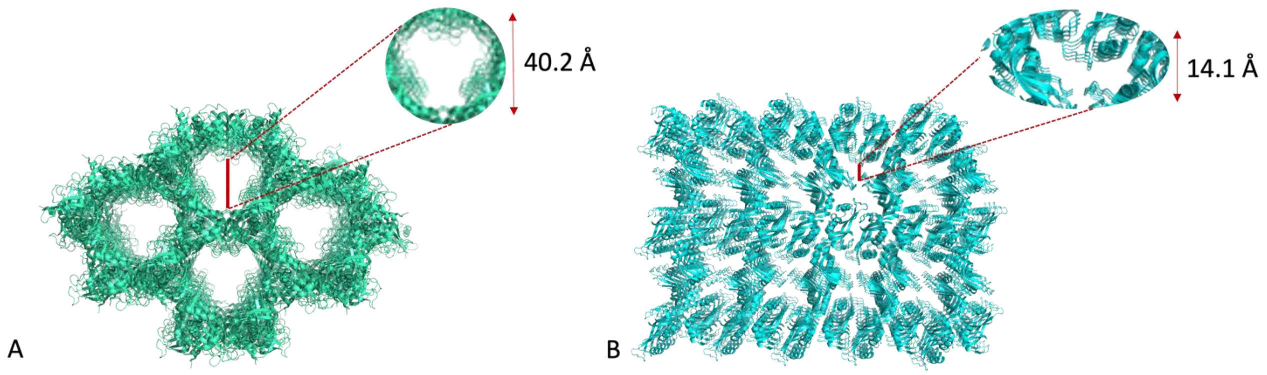

Figure S1. Porous structures in RNase A crystals. Crystal packing in the $P3_221$ space group showing a 4.0 nm porous channel (panel A, PDB code 5OGH)[1] and in the C2 space group showing a 1.4 nm porous channel (panel B, PDB code 1JVT)[2].

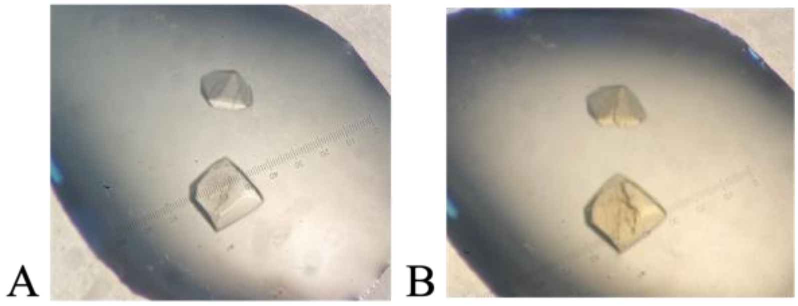

Figure S2. RNase A crystals grown in 2.5 M NaCl, 3.3 M sodium formate and 0.1 M sodium acetate pH 5.2 before (panel A) and after (panel B) GA treatment using gentle diffusion technique.

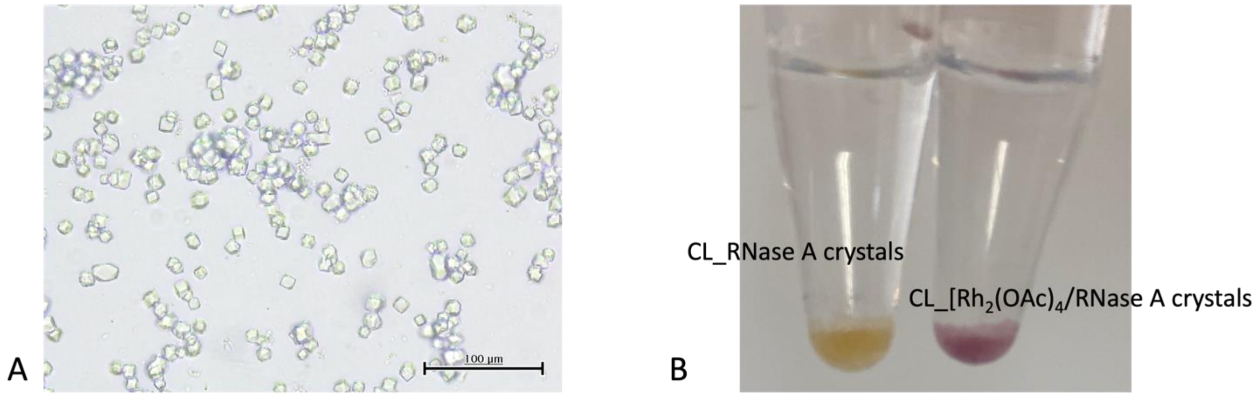

Figure S3. Picture of bulk RNase A crystals obtained using the batch technique (panel A) and photo of the crystals after GA treatment (CL_RNase A crystals) and after soaking with dirhodium tetraacetate (CL_[Rh$_2$(OAc)$_4$]/RNase A crystals) (panel B).

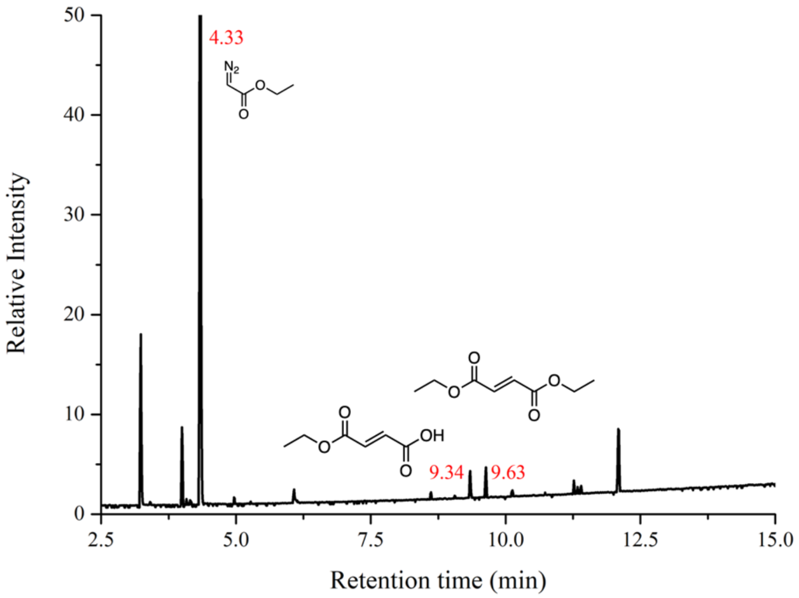

Figure S4 GC chromatogram of the reaction products obtained upon the self-coupling of diazo compounds reaction catalyzed by CL_[$Rh_2(OAc)_4$]/RNase A crystals. Labels in red indicate retention times of identified reaction products. The unlabelled peaks were not assigned. Peaks at t_R = 9.34 min and at t_R = 9.63 min were attributed to (E)-4-ethoxy-4-oxobut-2-enoic acid and diethyl fumarate, respectively.

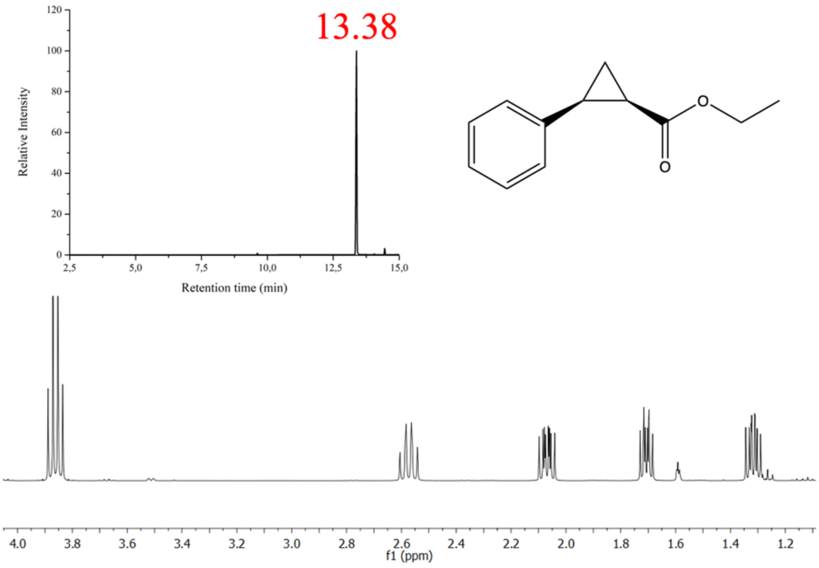

Figure S5. Close-up of ^{1}H-NMR spectrum of the *cis*-ethyl-phenylcyclopropane-1-carboxylate. A snapshot of the GC chromatogram of this compound is shown on the left.

cis-ethyl-phenylcyclopropane-1-carboxylate (400 MHz, solvent = $CDCl_3$). δ = 7.25 (m, 4H), 7.18 (m, 1H), 3.87 (q, 2H), 2.56 (dd, 1H), 2.06 (m, 1H), 1.70 (m, 1H), 1.30 (m, 1H), 0.95 (t, 3H) ppm. ^{1}H NMR spectra were recorded at 25 °C with a JNM-ECZL400 spectrometer (400 MHz, JEOL Ltd.) using $CDCl_3$ as solvent.

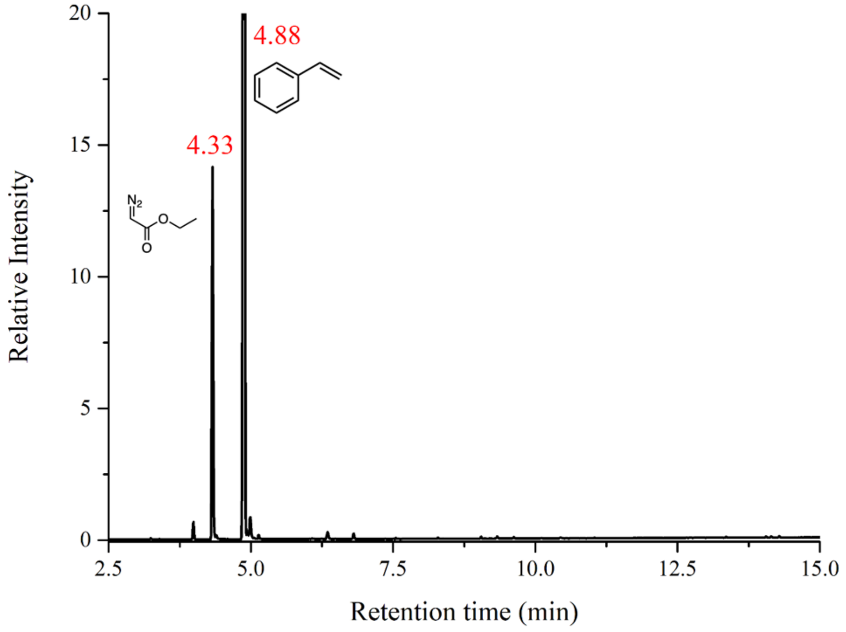

Figure S6. GC chromatogram used as control for the olefin cyclopropanation reaction. Styrene and ethyl diazoacetate were dissolved in a solution containing CL_RNase A crystals. Labels in red indicate retention times.

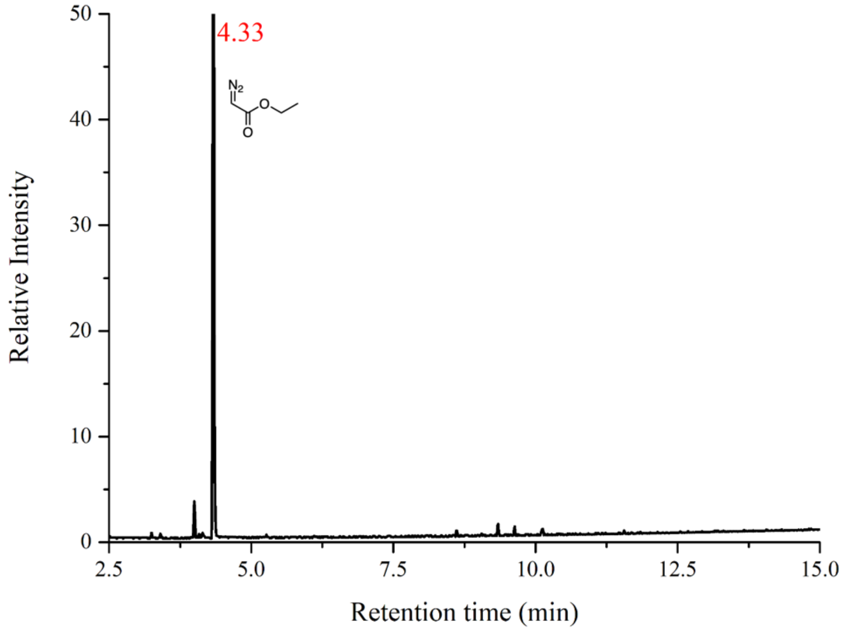

Figure S7. GC chromatogram used as control for the self-coupling of diazo compounds reaction. Ethyl diazoacetate was dissolved in a solution containing CL_RNase A crystals. Labels in red indicate retention times.

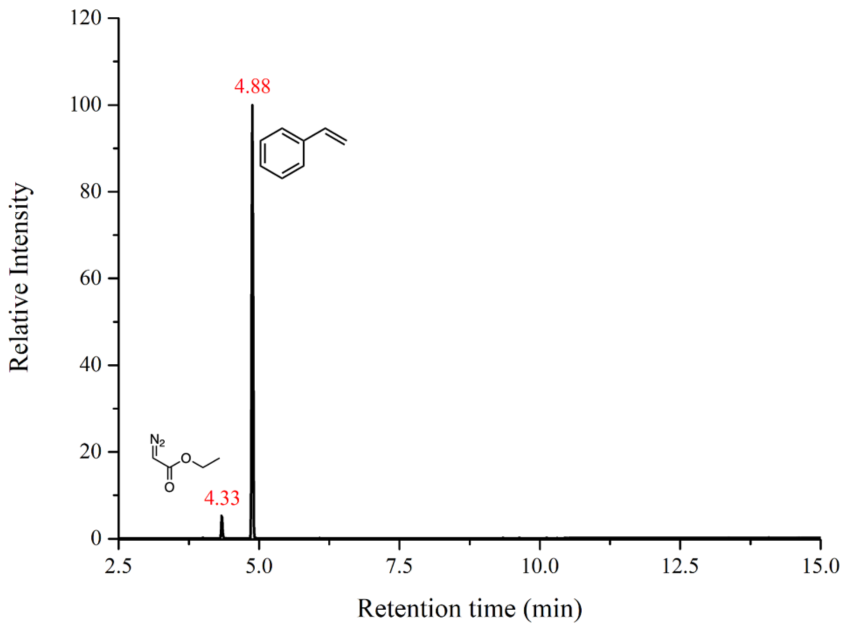

Figure S8. GC chromatogram of styrene and ethyl diazoacetate mixture.

Table S1. Rmsd obtained by superimposition of Cα atoms of the structures of CL_[$Rh_2(OAc)_4$]/RNase A crystals, [$Rh_2(OAc)_4$]/RNase A adduct crystals and the metal-free RNase A. -180 °C and 0 °C refer to the temperature used to collect X-ray diffraction data on CL_[$Rh_2(OAc)_4$]/RNase A crystals. Soaking time 1 h, soaking time 2 h and soaking time 6 h refer to the exposition time of RNase A crystals to [$Rh_2(OAc)_4$].

	[$Rh_2(OAc)_4$]/RNase A Soaking time 1 h (PDB code 8OQC)	[$Rh_2(OAc)_4$]/RNase A Soaking time 2 h (PDB code 8OQD)	[$Rh_2(OAc)_4$]/RNase A Soaking time 6 h (PDB code 8OQE)	CL_[$Rh_2(OAc)_4$]/RNase A Data collection temperature -180 °C (PDB code 8OQF)	CL_[$Rh_2(OAc)_4$]/RNase A Data collection temperature 0 °C (PDB code 8OQG)	Metal-free RNase A (PDB code 5OGH)
[$Rh_2(OAc)_4$]/RNase A Soaking time 1 h (PDB code 8OQC)	0	0.059	0.061	0.080	0.139	0.336
[$Rh_2(OAc)_4$]/RNase A Soaking time 2 h (PDB code 8OQD)		0	0.058	0.064	0.130	0.285
[$Rh_2(OAc)_4$]/RNase A Soaking time 6 h (PDB code 8OQE)			0	0.092	0.119	0.302
CL_[$Rh_2(OAc)_4$]/RNase A Data collection temperature -180 °C (PDB code 8OQF)				0	0.142	0.259
CL_[$Rh_2(OAc)_4$]/RNase A Data collection temperature 0 °C (PDB code 8OQG)					0	0.318
Metal-free RNase A (PDB code 5OGH)						0

Table S2. Selected bond lengths (Å) and angles (°) for the dirhodium centre coordinated to the side chains of His105 and His119 in the various structures reported in this paper. -180 °C and 0 °C refer to the temperature used to collect X-ray diffraction data on CL_[$Rh_2(OAc)_4$]/RNase A crystals. Soaking time 1 h, soaking time 2 h and soaking time 6 h refer to the exposition time of RNase A crystals to [$Rh_2(OAc)_4$].

Crystal		His119	His105
[$Rh_2(OAc)_4$]/RNase A Soaking time 1 h (PDB code 8OQC)	Rh—N (Å)	2.24	2.22
	Rh—Rh (Å)	2.37	2.40
	Rh—O_{OAc}[a] (Å)	2.06	2.10
	Rh—O_{wat}[a] (Å)	/	2.10
	Rh—Cl (Å)	2.55	2.61
	O_{OAc}—Rh—N[a] (°)	92.2	94.4
	N_{ax}—Rh—Rh(°)	179.3	176.6
	O_{wat}—Rh—Rh[a](°)	83.4	/
	Cl—Rh—Rh(°)	173.8	178.6
[$Rh_2(OAc)_4$]/RNase A Soaking time 2 h (PDB code 8OQD)	Rh—N (Å)	2.21	2.25
	Rh—Rh (Å)	2.38	2.38
	Rh—O_{OAc}[a] (Å)	2.11	2.04
	Rh—O_{wat}[a] (Å)	2.09	/
	Rh—Cl (Å)	2.56	2.58
	O_{OAc}—Rh—N[a] (°)	94.9	91.8
	N_{ax}—Rh—Rh(°)	173.4	178.7
	O_{wat}—Rh—Rh[a](°)	88.7	/
	Cl—Rh—Rh(°)	172.7	178.3
[$Rh_2(OAc)_4$]/RNase A Soaking time 6 h (PDB code 8OQE)	Rh—N (Å)	2.21	2.24
	Rh—Rh (Å)	2.37	2.38
	Rh—O_{OAc}[a] (Å)	2.09	2.05
	Rh—O_{wat}[a] (Å)	2.10	/
	Rh—Cl (Å)	2.63	2.57
	O_{OAc}—Rh—N[a] (°)	92.3	92.0

	N_{ax}—Rh—Rh(°)<br>O_{wat}—Rh—Rh[a](°)<br>Cl—Rh—Rh(°)	177.0<br>87.8<br>174.2	178.8<br>/<br>177.5
CL_[$Rh_2(OAc)_4$]/RNase A<br>Data collection temperature -180 °C<br>(PDB code 8OQF)	Rh—N (Å)<br>Rh—Rh (Å)<br>Rh—O_{OAc}[a] (Å)<br>Rh—O_{wat}[a] (Å)<br>Rh—Cl (Å)<br><br>O_{OAc}—Rh—N[a] (°)<br>N_{ax}—Rh—Rh(°)<br>O_{wat}—Rh—Rh[a] (°)<br>Cl—Rh—Rh(°)	2.35<br>2.50<br>2.12<br>2.10<br>/<br><br>109.5<br>168.9<br>105.2<br>/	2.27<br>2.38<br>2.06<br>/<br>2.59<br><br>92.2<br>179.7<br>/<br>178.9
CL_[$Rh_2(OAc)_4$]/RNase A<br>Data collection temperature 0 °C<br>(PDB code 8OQG)	Rh—N (Å)<br>Rh—Rh (Å)<br>Rh—O_{OAc}[a] (Å)<br>Rh—O_{wat}[a] (Å)<br>Rh—Cl (Å)<br><br>O_{OAc}—Rh—N[a] (°)<br>N_{ax}—Rh—Rh(°)<br>O_{wat}—Rh—Rh[a](°)<br>Cl—Rh—Rh(°)	2.17<br>2.46<br>/<br>2.10<br>/<br><br>/<br>169.7<br>91.4<br>/	2.25<br>2.38<br>2.03<br>2.62<br><br>92.1<br>178.2<br>/<br>178.4
[$Rh_2(OAc)_4$]/RNase A*<br>Space group C2.<br>Molecule A	Rh—N (Å)<br>Rh—Rh (Å)<br>Rh—O_{OAc}[a] (Å)<br>Rh—O_{wat}[a] (Å)<br><br>O_{OAc}—Rh—N[a] (°)<br>N_{ax}—Rh—Rh(°)	2.16<br>2.34<br>2.09<br>2.24<br><br>90.9<br>177.1	2.20<br>2.42<br>2.06<br>2.29<br><br>91.1<br>176.3
[$Rh_2(OAc)_4$]/RNase A*<br>Space group C2.<br>Molecule B	Rh—N (Å)<br>Rh—Rh (Å)<br>Rh—O_{OAc}[a] (Å)<br>Rh—O_{wat}[a] (Å)<br><br>O_{OAc}—Rh—N[a] (°)<br>N_{ax}—Rh—Rh(°)	2.27<br>2.37<br>2.11<br>2.28<br><br>91.9<br>175.4	2.18<br>2.42<br>2.13<br>2.28<br><br>93.3<br>176.6

[a]Average values. Standard deviations for the distances are in the range of 0.01-0.05 Å. Standard deviations for the angles are in the range of 0.6-7.1 °. 'wat' in the table refers only to water coordinating Rh atoms at equatorial positions.
*Values derived from reference [3].

References

(1) Prats-Ejarque, G.; Blanco, J. A.; Salazar, V. A.; Nogués, V. M.; Moussaoui, M.; Boix, E. Characterization of an RNase with Two Catalytic Centers. Human RNase6 Catalytic and Phosphate-Binding Site Arrangement Favors the Endonuclease Cleavage of Polymeric Substrates. *Biochimica et Biophysica Acta (BBA) - General Subjects* **2019**, *1863* (1), 105–117. https://doi.org/10.1016/j.bbagen.2018.09.021.

(2) Vitagliano, L.; Merlino, A.; Zagari, A.; Mazzarella, L. Reversible Substrate-Induced Domain Motions in Ribonuclease A. *Proteins* **2002**, *46*, 97–104. https://doi.org/10.1002/prot.10033.

(3) Ferraro, G.; Pratesi, A.; Messori, L.; Merlino, A. Protein Interactions of Dirhodium Tetraacetate: A Structural Study. *Dalton Trans.* **2020**, *49* (8), 2412–2416. https://doi.org/10.1039/C9DT04819G.
